# Delayed diagnosis of active pulmonary tuberculosis - potential risk factors for patient and healthcare delays in Portugal

**DOI:** 10.1186/s12889-021-12245-y

**Published:** 2021-11-27

**Authors:** João Almeida Santos, Andreia Leite, Patrícia Soares, Raquel Duarte, Carla Nunes

**Affiliations:** 1 NOVA National School of Public Health, Public Health Research Centre, Universidade NOVA de Lisboa, Avenida Padre Cruz, 1600-560 Lisbon, Portugal; 2 National Health Institute Dr. Ricardo Jorge, Avenida Padre Cruz, 1600-560 Lisbon, Portugal; 3 Comprehensive Health Research Center (CHRC), Universidade NOVA de Lisboa, Campo Mártires da Pátria, 1169-056 Lisbon, Portugal; 4 Chest Disease Center, Vila Nova de Gaia, Rua Conselheiro Veloso da Cruz, 4400-092 Vila Nova de Gaia, Portugal; 5 Faculdade de Medicina da Universidade do Porto, Alameda Prof. Hernâni Monteiro, 4200-319 Porto, Portugal

**Keywords:** Pulmonary tuberculosis, Patient delay, Healthcare delay, Total delay, Tuberculosis control, Public health

## Abstract

**Background:**

Early diagnosis and treatment of pulmonary tuberculosis (PTB) is essential for an effective control of the tuberculosis (TB) epidemic. Delayed diagnosis and treatment of TB increases the chance of complications and mortality for the patients, and enhances TB transmission in the population. Therefore, the aim of this study was to characterize patient, healthcare and total delay in diagnosing PTB and assess the effect of clinical and sociodemographic factors on the time until first contact with healthcare or reaching a PTB diagnosis.

**Methods:**

Retrospective cohort study that included active PTB patients notified in the National Tuberculosis Surveillance System (SVIG-TB), between 2008 and 2017. Descriptive statistics, Kaplan-Meier estimates, logrank test and Cox proportional hazards model were used to characterize patient, healthcare and total delay and estimate the effect of clinical and sociodemographic variables on these delays. Significance level was set at 0.05.

**Results:**

Median patient, healthcare and total delays was 37 days (Interquartile range (IQR): 19–71), 8 days (IQR: 1–32) and 62 days (IQR: 38–102), respectively.

The median patient delay showed a constant increase, from 33 days in 2008 to 44 days in 2017. The median total delay presented a similar trend, increasing from 59 days in 2008 to 70 days in 2017. Healthcare delay remained constant during the study period. More than half of the PTB cases (82.9%) had a delay > 1 month between symptom onset and diagnosis.

In the final Cox model, alcohol abuse, unemployment and being from a high TB incidence country were factors significantly associated with longer patient delay, while being female, having more than 45 years, oncologic and respiratory diseases were associated with longer healthcare delay. Being female, having more than 45 years and being from a high TB incidence country were associated with longer total delay.

**Conclusions:**

Patient delay and total delay have increased in recent years. Older patients, patients with alcohol problems, other comorbidities, unemployed or from countries with high TB incidence would benefit from the development of specific public health strategies that could help reduce the delay in TB diagnosis observed in our study.

This study emphasizes the need to promote awareness of TB in the general population and among the healthcare community, especially at ambulatory care level, in order to reduce the gap between beginning of symptoms and TB diagnosis.

**Supplementary Information:**

The online version contains supplementary material available at 10.1186/s12889-021-12245-y.

## Background

Tuberculosis (TB) remains one of the greatest public health challenges of our time. In 2019, an estimated 10.0 million people had TB and 1.4 million people died of the disease worldwide, making TB the main cause of mortality and morbidity through infection [1]. Portugal has seen a steady reduction in the number of notified TB cases during the last decade [2] but still has one of the highest TB incidence rates in the European Union (EU), only surpassed by some Eastern European countries [3]. Therefore, despite advances in the control of the TB epidemic on a global scale, the road is still long and fraught with challenges until TB elimination can be achieved as intended by the End TB Strategy, a strategy of the World Health Organization (WHO) to control and eliminate the TB epidemic by 2035 [4, 5].

TB usually presents with nonspecific symptoms that can easily be mistaken for other prevalent febrile infections, especially in the early stages of the disease. As early-stage symptoms are not usually disabling and allow to continue with day-to-day activities, the disease may progress for weeks or months before becoming severe enough for patients to seek care or be diagnosed with active TB [6]. During this time, people who come into close contact with the patient are at risk of being infected. 

Most TB control programs adopt the passive case finding as the predominant approach to detect TB cases, which means that cases are detected when people experience symptoms that lead them to seek the healthcare services [7]. Successful case detection also relies on health care systems capacity to quickly diagnose and initiate treatment [8]. Therefore, early diagnosis and prompt TB treatment is essential for an effective TB control program [9, 10] since delaying the diagnosis worsens illness severity, prolongs patient suffering, increases the risk of patient death, and enables the transmission within the community [8]. Delayed diagnosis and TB treatment occur in both high and low TB prevalence countries [8, 11].

These delays can be linked to both patient and the healthcare system. Patients can postpone seeking medical aid (patient delay) or the healthcare providers may not recognize and diagnose a TB case in a timely manner (healthcare delay) [11]. Although there is no universally accepted period for total delay (sum of patient and healthcare delays), it is generally accepted that total delay should not exceed one month for the majority of TB patients [12, 13].

There are several studies on TB diagnosis and treatment delay with the majority conducted in low and middle-income countries [11, 14–18], where the TB epidemic, socioeconomic and healthcare systems are substantially different from the high income countries. Several demographic, socioeconomic and clinical factors have been identified in these studies that may impact negatively the time to diagnosis and beginning of treatment [10, 11, 15, 16, 18–20], such as unfavorable social and economic conditions, accessibility or quality of services, education, age, gender, human immunodeficiency virus (HIV) co-infection or alcohol and drug addiction [8, 15].

Some studies [21–23] have already been carried out in Portugal that analyze the delay in diagnosing TB cases, but a broader notion of the evolution of delays over the years and a better understanding of the factors that influence delays, whether at the patient, healthcare system or global level, are needed. Identifying these factors could support the improvement of health services and assist in the development of strategies to prevent and control the spread of the disease more effectively.

Therefore, the aim of this study was to characterize patient, healthcare and total delay in diagnosing pulmonary TB (PTB) and assess the effect of clinical and sociodemographic factors on the time until first contact with healthcare or reaching a PTB diagnosis.

## Methods

### Study design and data source

Retrospective cohort study that used data from active PTB cases notified in the Portuguese National Tuberculosis Surveillance System (SVIG-TB). SVIG-TB is a clinical-based notification system in which the information is generated by direct collection of information from healthcare providers, who notify the case at the time of diagnosis and update the information during follow-up, to a network that aggregates the information at a national level [24].

### Study data

The study included all PTB cases notified in the SVIG-TB database between 2008 and 2017. Were excluded from the study patients that: (i) were not identified through passive case finding, as these patients did not seek medical care for their symptoms and were identified due to other reasons (e.g., contact tracing); (ii) had a total delay of more than one year (> 365 days), as it is unlikely that such a long delay would occur, suggesting the possibility of errors in the introduction of the dates that define the time until diagnosis, either when filling out the forms or when entering the information in the database; (iii) had absent or incorrect dates for symptoms onset, first healthcare contact and/or diagnosis, as it prevents establishing patient, healthcare and/or total delay for these patients.

For the present study, three outcomes were considered: patient delay, healthcare delay and total delay.

Patient delay was defined as the time between the date of onset of symptoms possibly related to PTB and the patient’s first medical appointment, and healthcare delay was defined as the period between the first medical appointment and the PTB diagnosis date [6, 11, 25]. Total delay was defined as the period between the date of symptoms onset and the diagnosis date (sum of patient and healthcare delays) [6, 11, 25].

The clinical and sociodemographic variables comprised in the analysis were selected based on the literature [11, 15, 23], and included sex (male/female); age at diagnosis; country of origin (Portugal/country of low TB incidence (< 20/100000 population)/country of high TB incidence (> 20/100000 population) [4, 26]); comorbidities [chronic renal failure on dialysis (yes/no), oncologic diseases (yes/no), inflammatory diseases (yes/no), respiratory diseases (yes/no), diabetes (yes/no) and HIV infection (yes/no)], substance abuse [alcohol abuse (yes/no) and drug abuse (yes/no)], homeless (yes/no); community residence (yes/no); unemployment (yes/no); health professional (yes/no).

### Statistical analysis

A descriptive analysis of the study population was performed using absolute and relative frequencies for categorical variables and median and interquartile range (IQR = Quartile 1 - Quartile 3) for numerical variables. The distribution of variables between groups (included and excluded) was compared using the chi-square test, as a way to assess selection bias. Total delay and its components were categorized into four groups: ≤1 month, between 1 and 2 months, 2 and 3 months and > 3 months

Three different analyses for each outcome (patient delay, healthcare delay and total delay) were performed. Since the event occurred for each patient, the study did not present censored times. To assess the effect of clinical and sociodemographic variables on the time until the event of interest (first contact with healthcare or PTB diagnosis), Kaplan-Meier estimator [27] and the logrank test [28] were used to compare the survival curves between groups in the same variable. Significant variables (*p*-value ≤0.05) were considered for the multivariable analyses.

Cox proportional hazards model [29] was used to estimate the effect of the clinical and sociodemographic variables on the time until the event of interest. All significant variables in the logrank test were initially included in the model. The final model was derived using a backward stepwise approach, keeping only variables with a p-value ≤0.05 in the final model.

Hazard ratios (HR) with 95% confidence intervals (95%CI) were used as the measure of association, with HR values > 1 constituting a higher risk of having the event per unit of time. In other words, HR > 1 represented a shorter time to approach the healthcare services (patient delay) or being diagnosed (healthcare and total delay), while HR < 1 represented a longer time to event and, therefore, higher patient, healthcare or total delays.

The proportional hazards assumption was assessed through the Schoenfeld residuals. The linear correlation between the residuals and time was tested and scatterplots were drawn to check the proportional hazards assumption.

All statistical analyses were performed using IBM® SPSS® Statistics for Windows, version 23 (IBM Corp., N.Y., USA).

## Results

### Population characterization

From 2008 to 2017, 15,359 cases of active PTB identified through passive case-finding were notified to SVIG-TB. 23.4% (*n* = 3598) of these cases were excluded from the analysis due to presenting more than 365 days from symptoms onset to diagnosis or due to inconsistencies in the dates for the beginning of symptoms, first healthcare contact or diagnosis, resulting in a final sample of 11,762 patients. When comparing the included and excluded individuals, there were some differences between the groups as shown in the Supplementary Table 1.

70.4% (*n* = 8281) of the patients included in the study were male, with mean age of 46.4 years (± standard deviation 17.9), ranging from less than 1 to 100 years, and with Portugal as the more frequent country of origin (*n* = 9901, 84.2%) (Table 1). More than half of the PTB cases (*n* = 6080, 51.7%) were notified in the districts of Lisbon (*n* = 3068, 26.1%) and Porto (*n* = 3012, 25.6%), the two main urban areas of the country (Supplementary Table 1).

**Table 1:** Patient, healthcare and total median delay, interquartile range (IQR) and logrank test (*p*-value) by clinical and sociodemographic variables

Variables	Patients	Patient delay		Healthcare delay		Total delay	
	n/N (%)	Median (IQR) (days)	Logrank (*p*-value)	Median (IQR) (days)	Logrank (*p*-value)	Median (IQR) (days)	Logrank (*p*-value)
Sex			0,395		< 0,001		< 0,001
Male	8281/11762 (70.4%)	37 (19–71)		7 (1–30)		61 (37–100)	
Female	3481/11762 (29.6%)	36 (19–70)		11 (2–39)		66 (40–106)	
Age			< 0,001		< 0,001		< 0,001
0–4 years	50/11750 (0.4%)	25.5 (17–49)		10.5 (4–44)		47 (32.5–95)	
5–14 years	73/11750 (0.6%)	33 (18–70)		7 (2–21.5)		52 (32–88)	
15–24 years	1109/11750 (9.4%)	36 (20–65)		6 (1–22)		55 (34–83)	
25–34 years	1951/11750 (16.6%)	39 (21–74)		7 (1–25)		59 (37–97)	
35–44 years	2682/11750 (22.8%)	38 (19–74)		6.5 (1–27)		60 (36–99)	
45–54 years	2430/11750 (20.7%)	40 (20–75)		7 (1–28)		64.5 (38–106)	
55–64 years	1464/11750 (12.4%)	38 (20–75)		10 (1–36)		68 (40–109)	
> = 65 years	1991/11750 (16.9%)	30 (14–60)		21 (4–55)		70 (41–112)	
Country of origin			< 0,001		0.002		0.033
Portugal	9901/11747 (84.2)	36 (18–69)		9 (1–33)		62 (37–102)	
Country of high TB incidence	1752/11747 (14.9)	44 (24–80)		7 (1–27)		66,5 (41–106)	
Country of low TB incidence	94/11747 (0.8)	34.5 (18–64)		8 (1–28)		59 (31–99)	
Comorbidities							
Chronic renal failure	100/11762 (0.9%)		< 0,001		0,013		0,316
Yes		24 (9–42)		21 (4–46)		52 (26–87)	
No		37 (19–71)		8 (1–32)		62 (38–102)	
Oncologic diseases	488/11762 (4.1%)		0,001		< 0,001		0,048
Yes		30 (15–55)		26 (5–55)		66 (42–108)	
No		37 (19–72)		8 (1–31)		62 (37–102)	
Inflammatory diseases	107/11572 (0.9%)		0,414		0,036		0,04
Yes		32 (16–83)		16 (2–48)		79 (42–117)	
No		37 (19–71)		8 (1–33)		62 (38–102)	
Respiratory diseases	647/11762 (5.5%)		0,231		< 0,001		0,004
Yes		33 (14–69)		13 (2–46)		70 (40–113)	
No		37 (19–71)		8 (1–32)		62 (37–102)	
Diabetes	780/11762 (6.6%)		0,929		0,003		0,087
Yes		38 (19–73)		11 (2–39)		68 (41–111)	
No		37 (19–71)		8 (1–32)		62 (37–102)	
HIV infection	1240/10793 (11.5%)		< 0,001		< 0,001		< 0,001
Yes		36 (19–64)		8 (1–27)		56 (35–89)	
No		38 (19–73)		8 (1–33)		64 (38–105)	
Substance abuse							
Alcohol abuse	1832/11270 (16.3%)		< 0,001		< 0,001		0,699
Yes		43 (22–81)		5 (1–21)		62.5 (36–103)	
No		36 (18–68)		9 (1–35)		62 (38–102)	
Drug abuse	1234/11363 (10.9%)		0,421		< 0,001		0,001
Yes		38 (19–74)		5 (1–23)		57 (34–95)	
No		37 (19–70)		9 (1–34)		63 (38–103)	
Homeless	201/11518 (1.7%)		0,745		< 0,001		0,001
Yes		39 (20–76)		4 (1–20)		54 (29–90)	
No		37 (19–71)		8 (1–33)		63 (38–103)	
Community residence	366/11479 (3.2%)		0,099		0,001		< 0,001
Yes		33 (19–60)		6 (1–22)		53 (33–86)	
No		37 (19–72)		8 (1–33)		63 (38–103)	
Unemployment	1891/11762 (16.1%)		< 0,001		< 0,001		0,357
Yes		42 (21–79)		5 (1–22)		61 (36–100)	
No		36 (18–70)		9 (1–34)		62 (38–103)	
Health professional	463/11762 (3.9%)		0,001		0,69		0,024
Yes		30 (16–59)		9 (1–33)		57 (31–98)	
No		37 (19–72)		8 (1–32)		62 (38–102)	

*IQR* interquartile range (Q1-Q3)

**Fig. 1:**
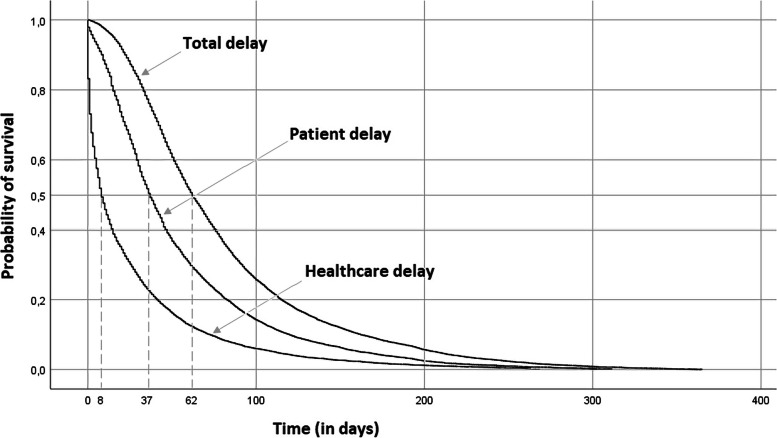
Survival curves for patient, healthcare and total delays for the 11,762 patients enrolled in the study (2008–2017)

The survival curves of patient, healthcare and total delays are shown in Fig. 1. The curves are similar in shape, initially with more patients reaching the event quickly and then decelerating over time. Healthcare delay presented the lowest median delay (8 days, IQR: 1–32 days), followed by patient delay (37 days, IQR: 19–71 days), and lastly by total delay (62 days, IQR: 38–102 days).

### Patient delay

**Fig. 2:**
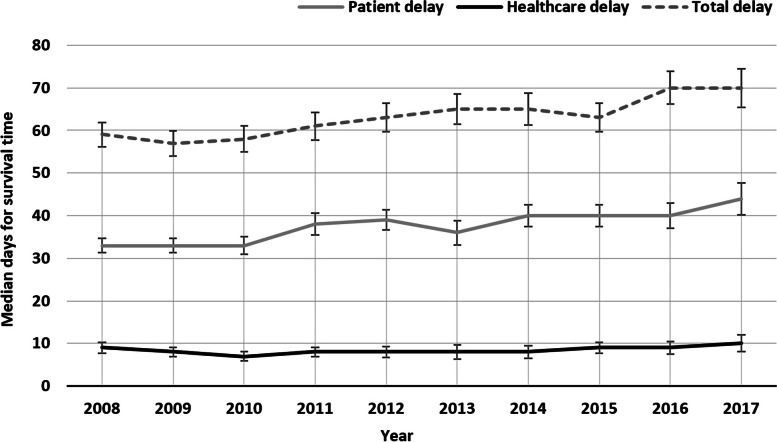
Annual median days for survival time with confidence intervals (95%CI) by patient, healthcare and total delay (2008–2017)

The median time between symptom onset and first healthcare contact presented an increasing trend over the years, ranging from 33 days in 2008 to 44 days in 2017 (11 days difference) (Fig. 2).

**Fig. 3:**
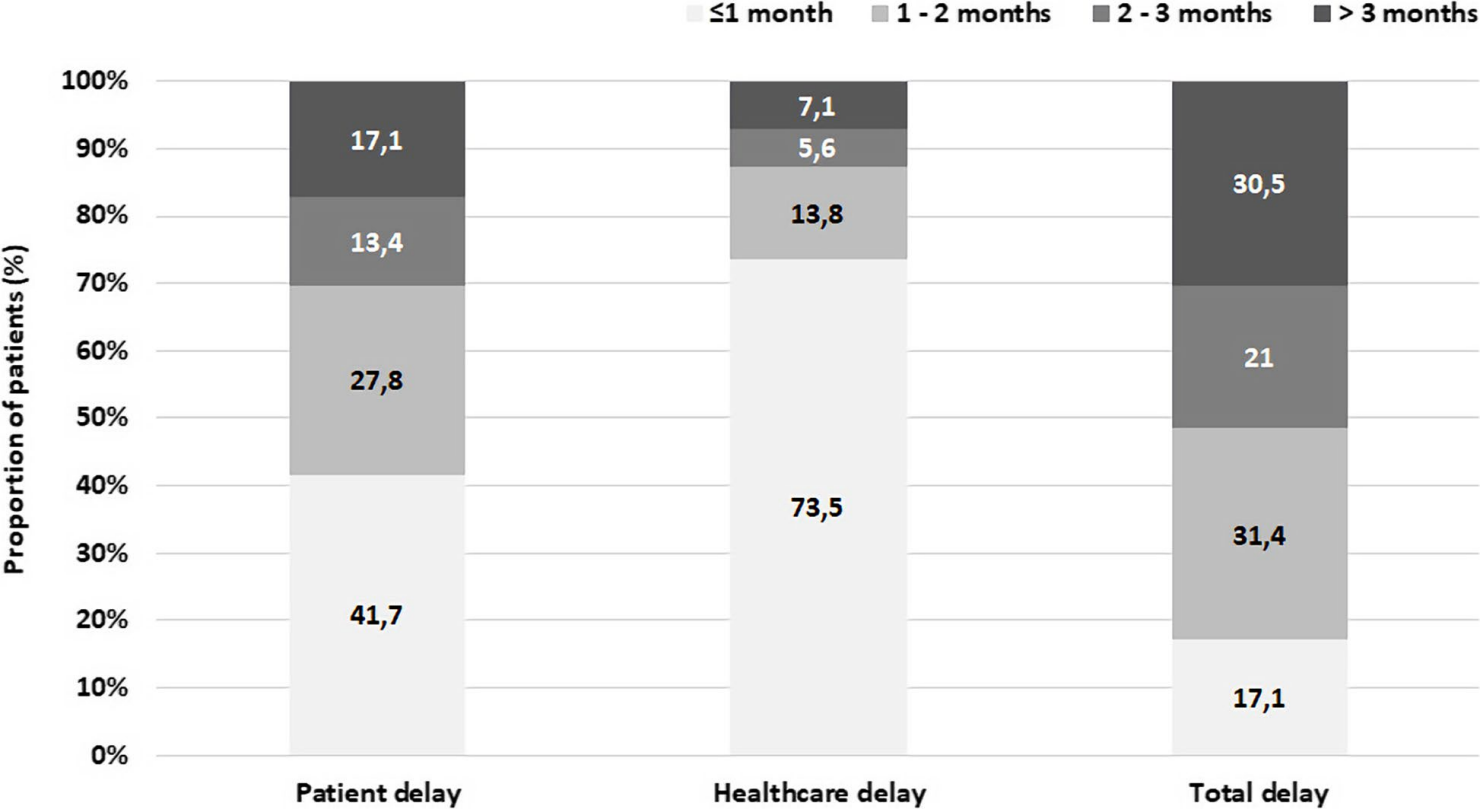
Proportion of patients who sought the healthcare services (patient delay), or were diagnosed (healthcare delay and total delay) within 1 month, between 1 and 2 months, 2 and 3 months and > 3 months

Nearly half of the patients (41.7%, *n* = 4907) had their first healthcare contact within 1 month after symptom onset, while more than a quarter of the patients took more than 3 months to seek help after symptom onset (17.1%, *n* = 2009) (Fig. 3).

Country of origin, chronic renal failure, oncologic diseases, HIV infection, alcohol abuse, unemployment and health professionals exhibited statistically significant differences between the survival curves of the groups in each variable. Patients from a high TB incidence country, with alcohol problems or unemployed presented higher median patient delays than patients without these risk factors. On the other hand, patients with chronic renal failure, oncological diseases, HIV infection or health professionals presented a lower median patient delay than patients without these risk factors.

**Fig. 4:**
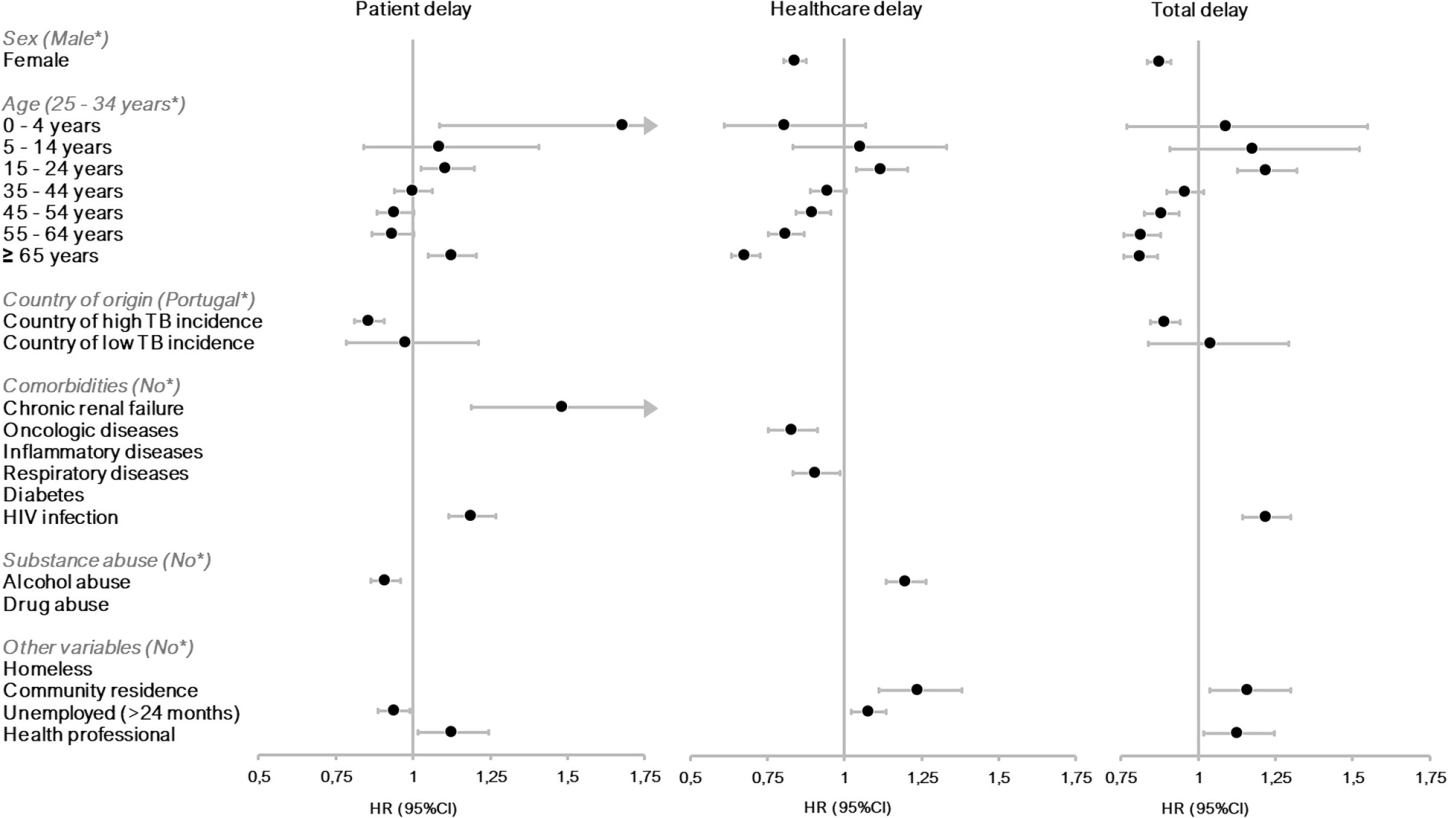
Hazard ratios (HR) with confidence intervals (95%CI) for the variables included in the final multivariate Cox proportional hazard model for patient, healthcare and total delay. *Reference category. HR values> 1 indicate a greater risk of having the event per unit of time, therefore a shorter time until approaching the healthcare services (patient delay) or diagnosis (healthcare and total delay)

In the final multivariate Cox proportional hazard model, country of origin [country of high TB incidence (0.858) - Reference category: Portugal], alcohol abuse (HR 0.91) and unemployment (HR 0.938) presented a statistically significant association with longer patient delay (HR < 1) (Fig. 4 and Table 2). Age [0–4 (HR 1.681), 15–24 (HR 1.107), ≥65 years (HR 1.124) - Reference category: 25–34], chronic renal failure (HR 1.485), HIV infection (HR 1.188) and being a health professional (HR 1.124) presented a statistically significant association with a shorter delay time.

**Table 2:** Hazard ratios and confidence intervals for the variables included in the final multivariate Cox proportional hazard model by patient, healthcare and total delay

	Patient delay (Final model)		Healthcare delay (Final model)		Total delay (Final model)	
Variables	HR (95%CI)	p-value	HR (95%CI)	p-value	HR (95%CI)	*p*-value
Sex (Male^a^)						
Female			0.839 (0.804–0.875)	< 0.001	0.874 (0.837–0.913)	< 0.001
Age group (25–34 years^a^)						
0–4 years	1.681 (1.184–2.385)	0.004	0,807 (0.61–1.07)	0.136	1091 (0.769–1.549)	0.624
5–14 years	1.087 (0.84–1.406)	0.527	1052 (0.831–1.332)	0.674	1.177 (0.909–1.523)	0.216
15–24 years	1.107 (1.025–1.197)	0.01	1.118 (1.037–1.206)	0.004	1.219 (1.128–1.318)	< 0.001
35–44 years	0.999 (0.938–1.063)	0.965	0.946 (0.89–1.005)	0.074	0,957 (0.9–1.018)	0.166
45–54 years	0.94 (0.882–1.002)	0.059	0.896 (0.842–0.955)	0.001	0.881 (0.827–0.938)	< 0.001
55–64 years	0.932 (0.866–1.003)	0.059	0.808 (0.753–0.868)	< 0.001	0.817 (0.76–0.879)	< 0.001
> = 65 years	1.124 (1.049–1.204)	0.001	0.678 (0.634–0.725)	< 0.001	0.813 (0.76–0.87)	< 0.001
Country of origin (Portugal^a^)						
Country of high TB incidence	0.858 (0.811–0.907)	< 0.001			0.893 (0.845–0.943)	< 0.001
Country of low TB incidence	0.975 (0.784–1.213)	0.821			1.041 (0.838–1.293)	0.719
Comorbidities (Yes/No^a^)						
Chronic renal failure	1.485 (1.19–1.852)	< 0.001				
Oncologic diseases			0.829 (0.754–0.911)	< 0.001		
Inflammatory diseases						
Respiratory diseases			0.905 (0.833–0.984)	0.02		
Diabetes						
HIV infection	1.188 (1.114–1.267)	< 0.001			1.219 (1.144–1.299)	< 0.001
Substance abuse (Yes/No^a^)						
Alcohol abuse	0.91 (0.862–0.961)	0.001	1.197 (1.134–1.264)	< 0.001		
Drug abuse						
Homeless (Yes/No^a^)						
Community residence (Yes/No^a^)			1.239 (1.11–1.38)	< 0.001	1.161 (1.039–1.298)	0.008
Unemployment (Yes/No^a^)	0.938 (0.888–0.991)	0.022	1.077 (1.021–1.135)	0.006		
Health professional (Yes/No^a^)	1.124 (1.015–1.246)	0.025			1.126 (1.018–1.246)	0.022

^a^Reference category, *HR* Hazards ratio, *CI* confidence interval

HR > 1 indicate a higher risk of having the event per unit of time, i.e., a shorter time until approaching the healthcare services (patient delay) or diagnosis (healthcare and total delay)

Regarding the Schoenfeld residuals, the correlation between the residuals and time were tested and found that some variables violated the assumption of non-proportionality. However, even small violations will be significant given the large sample size. After visualization of the respective scatterplot to inspect the fit of the proportional hazard assumption, it was considered that the assumption was not violated as the plots do not show clear patterns or systematic deviations through time (Supplementary Fig. 1).

### Healthcare delay

The median time between first healthcare contact and diagnosis was relatively constant between 2008 and 2017, increasing one day during this period (2008–9 days, 2017–10 days) (Fig. 2).

Most of the patients (73.5%, *n* = 8650) had a diagnosis within 1 month after first healthcare contact, with only 7.1% (*n* = 837) being diagnosed more than 3 months after seeking medical aid (Fig. 3).

Healthcare delay presented the highest number of risk factors with significant differences between the survival curves of their subgroups, with being a health professional the only variable that did not present a statistically significant difference (*p* = 0.69) - Table 1. Female patients, Portugal-born patients, patients with chronic renal failure, diabetes, oncological, inflammatory or respiratory diseases presented higher median healthcare delays than patients without these risk factors. In contrast, patients with alcohol or drug problems, homeless, residing in a community residence, or unemployed presented a lower median healthcare delay than patients without these risk factors.

In the final multivariate Cox model, sex [female (HR 0.839) - Reference category: male], age ≥ 45 years, oncologic diseases (HR 0.829) and respiratory diseases (HR 0.905) were identified as presenting a statistically significant association with a longer healthcare delay (Fig. 4 and Table 2). Conversely, age [15–24 years (HR 1.118) - Reference category: 25–34], alcohol abuse (HR 1.197), community residence (HR 1.239) and unemployment (HR 1.077) were associated with a shorter healthcare delay.

Regarding the Schoenfeld residuals, was found that some variables violated the assumption of non-proportionality. However, after visual inspection it was considered that the assumption was not violated (Supplementary Fig. 2).

### Total delay

Median total delay, as observed in patient delay, presented a steady increase between 2008 and 2017 that resulted in 11 days increase during this period (2008–59 days, 2017–70 days) (Fig. 2).

**Fig. 5:**
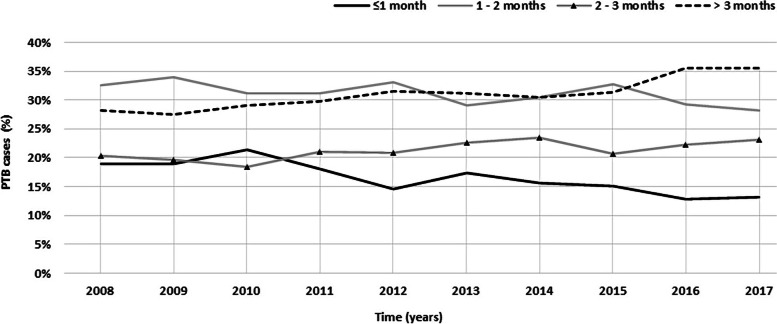
Annual proportion of patients diagnosed within 1 month, between 1 and 2 months, 2 and 3 months and > 3 months after symptoms onset

Just over a quarter of the patients (17.1%, *n* = 2012) were diagnosed within 1 month after symptom onset, while more than half (51.5%, *n* = 6054) were diagnosed more than 2 months after symptom onset (Fig. 3). Over the years, the proportion of patients with a total delay of more than 3 months has gradually increased while patients with a delay of less than 1 month have been decreasing (Fig. 5).

Sex, age, country of origin, oncologic diseases, inflammatory diseases, respiratory diseases, HIV infection, drug abuse, homeless, community residence and being a health professional exhibited statistically significant differences between the survival curves of their subgroups (Table 1). Female patients, patients from a high TB incidence country, patients with oncological, inflammatory or respiratory diseases presented higher median total delays than patients without these risk factors. On the contrary, patients with drug problems, homeless, residing in a community residence, or health professionals presented a lower median total delay than patients without these risk factors.

In the final multivariate Cox model, sex [female (HR 0.874) - Reference category: male], age ≥ 45 years and country of origin [country of high TB incidence (0.893) - Reference category: Portugal] presented a statistically significant association with a longer total delay (Fig. 4 and Table 2). Age [15–24 years (HR 1.219) - Reference category: 25–34], HIV infection (HR 1.219), community residence (HR 1.161) and being a health professional (HR 1.126) presented a statistically significant association with a shorter total delay.

Regarding the Schoenfeld residuals, some variables violated the assumption of non-proportionality. However, after visual inspection it was considered that the assumption was not violated as the plots do not show clear patterns or systematic deviations through time (Supplementary Fig. 3).

## Discussion

In our study, median healthcare delay was 8 days, a lower value than the one exhibited by the patient delay (37 days), with total delay presenting a median delay of 62 days between symptom onset and diagnosis. These results for patient and total delays are close to those obtained in a systematic review that summarized delays from low and high-income countries [11], however we observed a shorter healthcare delay (8 vs 21.5 days). This difference between healthcare delays may reflect the heterogeneity of the healthcare systems associated with the studies included in the systematic review. In Portugal, a study that evaluated the delays in pulmonary and extrapulmonary TB cases notified between 2010 and 2014 [23], also revealed similar values for median patient delay (33 days) and total delay (68 days) but the median healthcare delay was double of ours (17 days). Loutet et al. [30] established that healthcare delay was significantly longer among patients with extrapulmonary TB than those with pulmonary disease, since diagnosis of extrapulmonary TB is often delayed or overlooked due to misleading clinical presentation or poor performance of available diagnostic tests [31]. Thus, the contrast in median healthcare delays between the studies may reflect the inclusion of extrapulmonary TB cases in the analysis. Nevertheless, our results support those obtained in studies from other countries, where median patient delay is consistently higher than the healthcare delay [20, 30, 32–34].

Our results also show that Portugal is far from reaching the ideal time of 3 to 4 weeks [12] between the symptom onset and diagnosis (62 days vs 30 days). More than half of the cases of PTB (82.9%) notified in Portugal between 2008 and 2017 had a delay of more than 1 month between symptoms onset and diagnosis, with the proportion of patients that were diagnosed more than 3 months after symptoms onset displaying an increase trend during this period. These results are reflected in the median patient and total delays steady increase over the years, resulting in an increase of 11 days between 2007 and 2018. Healthcare delay remained relatively constant, however displaying an increase of 1 day between 2007 and 2018.

These findings suggest that delaying seeking help from the healthcare providers is the driving force behind the delay in diagnosing and beginning of treatment. Longer delays between symptoms onset and reaching for help from the healthcare services as an impact at an individual level, by increasing the patient risk of morbidity and mortality, and at a population level, by increasing the risk of spreading the disease through the community. Bello et al. [6] systematic review also shown a considerable delay in patients seeking help from the healthcare services. This delay can occur as a consequence of different factors such as the time it takes the patients to realize the symptoms and understand that they are ill, self-medication, needing time to think about seeking help from a health professional, or being able to overcome social, personal and physical barriers to obtain the necessary care [6, 33, 35]. Thus, in order to improve the control of the TB epidemic and reduce the time between the symptoms onset and TB diagnosis, strategies with an impact on the patient’s delay should be considered. However, these strategies should not be directed only at the patient (e.g., increase literacy about TB) but also directed to the healthcare system, and how the healthcare services and professionals can help patients reach the healthcare system in a shorter time [11, 12, 15].

Several clinical and sociodemographic factors were associated with delayed diagnosis, which could affect the patient, healthcare and/or total delay. Female patients experienced a significant longer healthcare and total delay when compared to male patients, as observed in other similar studies [30, 32, 36–38]. However, some studies have reached the opposite conclusions [14, 39] and others reported no statistically significant association between sex and late TB diagnosis and treatment [10, 20, 40]. These discrepancies may reflect differences in health seeking behaviors and gender roles between the different countries. PTB was investigated more readily in male patients, although female patients did not seek care later than men. One explanation could be that male patients are considered to have a higher risk of developing TB than female patients, currently the proportion of male:female TB cases is 2:1 [4], which may lead to a greater suspicion of TB in male patients with suggestive symptoms and, consequently, an earlier diagnosis. Further studies should be developed in order to understand the reasons for the delay in recognizing the disease in women, in order to establish strategies aimed at reducing the time between the first consultation and the diagnosis.

Age was another risk factor for longer healthcare and total delays, with the likelihood of longer delays increasing as age progressed. Patients over 45 years presented a significant association with a longer time between first contact with the healthcare system and a TB diagnosis. Other studies have also found this association [20, 30, 32, 33, 41], where older patients were more commonly misdiagnosed than younger patients. Despite not having significant delays in terms of seeking help from the healthcare services, older patients may present greater frequency of intercurrent or co-existing diseases or comorbidities that may decrease the suspicion of a TB diagnosis or make it more difficult to recognize TB symptoms [20, 30, 32]. Also, elderly patients often do not present with typical TB symptoms [10, 32, 42, 43], leading to older patients deteriorate without TB being considered and becoming a source of transmission in the community [44]. Harris et al. [45] in their study assessed the consequences of a long delay in TB diagnosis among elderly patients, which increased the likelihood of transmission and enhanced the severity of the TB disease and of the co-morbidities. Therefore, increased knowledge and awareness of TB, especially among professionals working closely with older people could help reduce transmission and also morbidity and mortality caused by the disease.

Foreign-born TB cases, more specifically from countries with a high TB incidence, were more likely to have a delay in accessing healthcare services when compared with Portugal-born patients. Migrants’ social, legal and economic circumstances can have a detrimental effect on accessing the healthcare system. Language barriers, organizational challenges, fear of immigration authorities, unfavorable socioeconomic conditions, poor awareness of symptoms and stigma [46, 47] are some of the difficulties that migrants face and that may justify a longer time to seek help from the healthcare service. On the other hand, Portugal-born patients experienced longer healthcare delays than foreign-born patients did. Possibly because healthcare professionals will have a higher index of suspicion of TB in patients with suggestive symptoms that are from countries with high TB incidence than Portugal-born, resulting in shorter healthcare delays. This finding is consistent with studies carried out in other countries low incidence countries [20, 48, 49].

Alcohol abuse and unemployment presented an association with longer delays, more specifically between beginning of symptoms and seeking help from the healthcare services. Previous studies [23, 50, 51] have established that patients with addictions are less likely to visit the healthcare services in a timely manner. This may result from the fact that patients who consume alcohol in excess may not seek, or easily have access to, healthcare providers when they start presenting symptoms and realize they are ill, resulting in delayed diagnosis and more advanced disease [52]. Notwithstanding, when these patients are first seen by a healthcare professional, the time to diagnosis is lower because of a high suspicion index [23]. Regarding unemployment, other studies have also shown that unemployment is an risk factor for delay in seeking treatment [18, 53, 54], as these patients are discourage by their economic situation from seeking healthcare in due time. Furthers studies should be conducted to support the design of interventions aimed at reducing the delay of these patients in reaching the healthcare system.

In our study, oncologic and respiratory diseases were associated with a longer healthcare delay. The coexistence of other diseases may delay the TB diagnosis, since they may have similar symptoms or even alter the typical symptoms indicative of TB. For example, sarcoidosis is a granulomatous disease with a similar clinical, radiological and histologic presentation as TB, making it difficult to identify a concurrent TB infection [55]. In addition, the non-specific nature of TB signs and symptoms, combined with the low TB incidence in Portugal, may lead to think to other causes such as pneumonia, bronchitis or asthma before TB.

Our study presents some limitations. Due to the large number of inconsistent or missing data, we were unable to calculate the patient, healthcare and/or total delay for 23.4% of the notified PTB patients, resulting in their exclusion from the analysis. Analyzing the differences between included and excluded patients (Supplementary Table 1), significant differences were identified between these patients. Excluded patients tended to be older, presenting a higher proportion of several comorbidities (oncologic and respiratory diseases, HIV infection) and disadvantaged socioeconomic conditions (homeless and residing in a community residence). Considering that some variables were associated with longer patient, healthcare and/or total delay, it is difficult to ascertain whether excluding these patients might have led to an under or overestimation of the diagnostic delay and, consequently, biased some of the identified associations. However, given that the differences between groups were small, we believe that this did not substantially influence our results. Furthermore, it is important to be careful when interpreting these results, as large sample sizes tend to produce smaller *p*-values. The presence of variables based on patient self-report (e.g., alcohol and drug abuse) may have led to a less accurate estimate of the impact of these variables in the delays. The date of symptoms onset was attained retrospectively, this fact may have caused patient delays to be affected by memory bias.

However, our analysis has different strengths. The study involved a considerable number of patients with different clinical and sociodemographic characteristics giving a broad and extensive view of the population delays, hoping to contribute significantly to a better understanding of patient, health and total delays in Portugal. The separate analysis of the patient and healthcare delays allowed us to observe whether the different risk factors included in the analysis affect each of the delays differently. This allowed us to recognize that the factors affect each of these dimensions differently, relevant information for the development of more targeted strategies to reduce each of the delays.

## Conclusion

Despite the improvement in the Portuguese health service over the last decades, the time between symptom onset and a TB diagnosis has increased over the years, furthering the transmission and occurrence of new cases of infection in the community and increasing the probability of disease progression, morbidity and mortality of infected patients.

In the present study, we observed that the time between symptom onset and first contact with the health system (patient delay) has increased over the years. This result highlights the need to develop strategies to reduce patient delay, such as improving health literacy, especially concerning TB, and emphasizing the importance of seeking healthcare services in a timely manner.

Several factors associated with delayed diagnosis were identified, some associated with a longer patient delay (alcohol abuse, unemployment and being from a high TB incidence country), while others with a longer healthcare delay (being female, having more than 45 years, oncologic and respiratory diseases). Identifying these factors allows the development of targeted public health policies and strategies, reaching population groups that present a significant delay in the diagnosis of TB and, consequently, help reduce the chain of transmission. One example is the implementation of policies and strategies to facilitate health care access to foreign-born patients, which would have benefits for both the potentially marginalized communities and the general population.

## Supplementary Information

**Additional file 1: Supplementary Table 1.** Sociodemographic and clinical characteristics of patients included and excluded in the analysis.

**Additional file 2: Supplementary Figure 1.**  Schoenfeld residues for the variables in the final Cox model - Patient delay. A - Country of origin, B - Age group, C - Health professional, D - Chronic renal failure, E - Alcohol abuse, F - HIV infection, G - Unemployment.

**Additional file 3: Supplementary Figure 2.** Schoenfeld residues for the variables in the final Cox model - Healthcare delay. A - Sex, B - Age group, C - Alcohol abuse, D - Oncologic diseases, E - Respiratory diseases, F - Community residence, G - Unemployment.

**Additional file 4: Supplementary Figure 3.**  Schoenfeld residues for the variables in the final Cox model - Total delay. A - Sex, B - Age group, C - Health professional, D - HIV infection, E - Country of origin, F - Community residence.

## Data Availability

The dataset supporting the conclusions of this article was obtained through the Portuguese National Tuberculosis Surveillance System (SVIG-TB) and is not publicly available. The SVIG-TB is supervised by the Directorate General of Health (Direção Geral da Saúde, DGS) that grants access to the fully anonymized dataset for epidemiological studies. The aggregated dataset used in this specific study is available from the corresponding author upon reasonable request.

## Abbreviations

95%CI: 95% confidence intervals
CI: Confidence Interval
EU: European Union
HIV: Human immunodeficiency virus
HR: Hazard ratios
IQR: Interquartile range
PTB: Pulmonary Tuberculosis
Q: Quartile
SVIG-TB: National Tuberculosis Surveillance System
TB: Tuberculosis
WHO: World Health Organization
