# Supplementary material for: Delayed diagnosis of active pulmonary tuberculosis - potential risk factors for patient and healthcare delays in Portugal
Source: BMC Public Health. 2021 Nov 27;21:2178. doi: 10.1186/s12889-021-12245-y (PMC8627051; doi:10.1186/s12889-021-12245-y)
Supplement: Supplementary file 1 — Additional file 1: Supplementary Table 1. Sociodemographic and clinical characteristics of patients included and excluded in the analysis. [file 12889_2021_12245_MOESM1_ESM.docx]

| **Supplementary Table 1.** Sociodemographic and clinical characteristics of patients included and excluded in the analysis. | | | |
| --- | --- | --- | --- |
| Variables | Patients | | χ²  (p-value) |
|  | Included | Excluded |  |
|  | n/N (%) | n/N (%) |  |
| Total | 11762 (76.6) | 3597 (23.4) |  |
| Sex |  |  | 0.139 |
| Male | 8281/11762 (70.4%) | 2486/3597 (69.1%) |  |
| Female | 3481/11762 (29.6%) | 1111/3597 (30.9%) |  |
| Age |  |  | <0.001 |
| 0 - 4 years | 50/11750 (0.4%) | 20/3589 (0.6%) |  |
| 5 - 14 years | 73/11750 (0.6%) | 32/3589 (0.9%) |  |
| 15 - 24 years | 1109/11750 (9.4%) | 305/3589 (8.5%) |  |
| 25 - 34 years | 1951/11750 (16.6%) | 480/3589 (13.3%) |  |
| 35 - 44 years | 2682/11750 (22.8%) | 718/3589 (20.0%) |  |
| 45 - 54 years | 2430/11750 (20.7%) | 697/3589 (19.4%) |  |
| 55 - 64 years | 1464/11750 (12.4%) | 544/3589 (15.1%) |  |
| >= 65 years | 1991/11750 (16.9%) | 793/3589 (22.0%) |  |
| Districts |  |  | <0.001 |
| Aveiro | 792/11762 (6.7%) | 134/3597 (3.7%) |  |
| Açores Autonomous Region | 47/11762 (0.4%) | 74/3597 (2.1%) |  |
| Beja | 158/11762 (1.3%) | 50/3597 (1.4%) |  |
| Braga | 828/11762 (7%) | 206/3597 (5.7%) |  |
| Bragança | 119/11762 (1%) | 4/3597 (0.1%) |  |
| Castelo branco | 143/11762 (1.2%) | 50/3597 (1.4%) |  |
| Coimbra | 89/11762 (0.8%) | 176/3597 (4.9%) |  |
| Évora | 67/11762 (0.6%) | 11/3597 (0.3%) |  |
| Faro | 500/11762 (4.3%) | 276/3597 (7.7%) |  |
| Guarda | 108/11762 (0.9%) | 22/3597 (0.6%) |  |
| Leiria | 236/11762 (2%) | 83/3597 (2.3%) |  |
| Lisboa | 3068/11762 (26.1%) | 1021/3597 (28.4%) |  |
| Madeira Autonomous Region | 146/11762 (1.2%) | 8/3597 (0.2%) |  |
| Portalegre | 103/11762 (0.9%) | 10/3597 (0.3%) |  |
| Porto | 3012/11762 (25.6%) | 1045/3597 (29.1%) |  |
| Santarém | 289/11762 (2.5%) | 142/3597 (3.9%) |  |
| Setúbal | 1233/11762 (10.5%) | 161/3597 (4.5%) |  |
| Viana do castelo | 325/11762 (2.8%) | 29/3597 (0.8%) |  |
| Vila real | 239/11762 (2%) | 13/3597 (0.4%) |  |
| Viseu | 260/11762 (2.2%) | 82/3597 (2.3%) |  |
| Country of origin |  |  | 0.115 |
| Portugal | 9901/11747 (84.2) | 3034/3568 (84.3) |  |
| Country of high TB incidence | 1752/11747 (14.9) | 496/3568 (13.8) |  |
| Country of low TB incidence | 94/11747 (0.8) | 38/3568 (1.1) |  |
| Comorbidities |  |  |  |
| Chronic renal failure | 100/11762 (0.9%) | 36/3597 (1.0%) | 0.399 |
| Oncologic diseases | 488/11762 (4.1%) | 236/3597 (6.6%) | <0.001 |
| Inflammatory diseases | 107/11572 (0.9%) | 34/3488 (0.9%) | 0.788 |
| Respiratory diseases | 647/11762 (5.5%) | 258/3597 (7.2%) | <0.001 |
| Diabetes | 780/11762 (6.6%) | 233/3597 (6.5%) | 0.745 |
| HIV infection | 1240/10793 (11.5%) | 408/3037 (13.4%) | <0.001 |
| Substance abuse |  |  |  |
| Alcohol abuse | 1832/11270 (16.3%) | 448/3257 (13.8%) | <0.001 |
| Drug abuse | 1234/11363 (10.9%) | 365/3301 (11.1%) | <0.001 |
| Homeless | 201/11518 (1.7%) | 63/3354 (1.9%) | <0.001 |
| Community residence | 366/11479 (3.2%) | 135/3345 (4.0%) | <0.001 |
| Unemployment | 1891/11762 (16.1%) | 511/3597 (14.2%) | 0.007 |
| Health professional | 463/11762 (3.9%) | 131/3597 (3.6%) | 0.423 |
