# Supplementary figures and images for: Delayed diagnosis of active pulmonary tuberculosis - potential risk factors for patient and healthcare delays in Portugal
Source: BMC Public Health. 2021 Nov 27;21:2178. doi: 10.1186/s12889-021-12245-y (PMC8627051; doi:10.1186/s12889-021-12245-y)

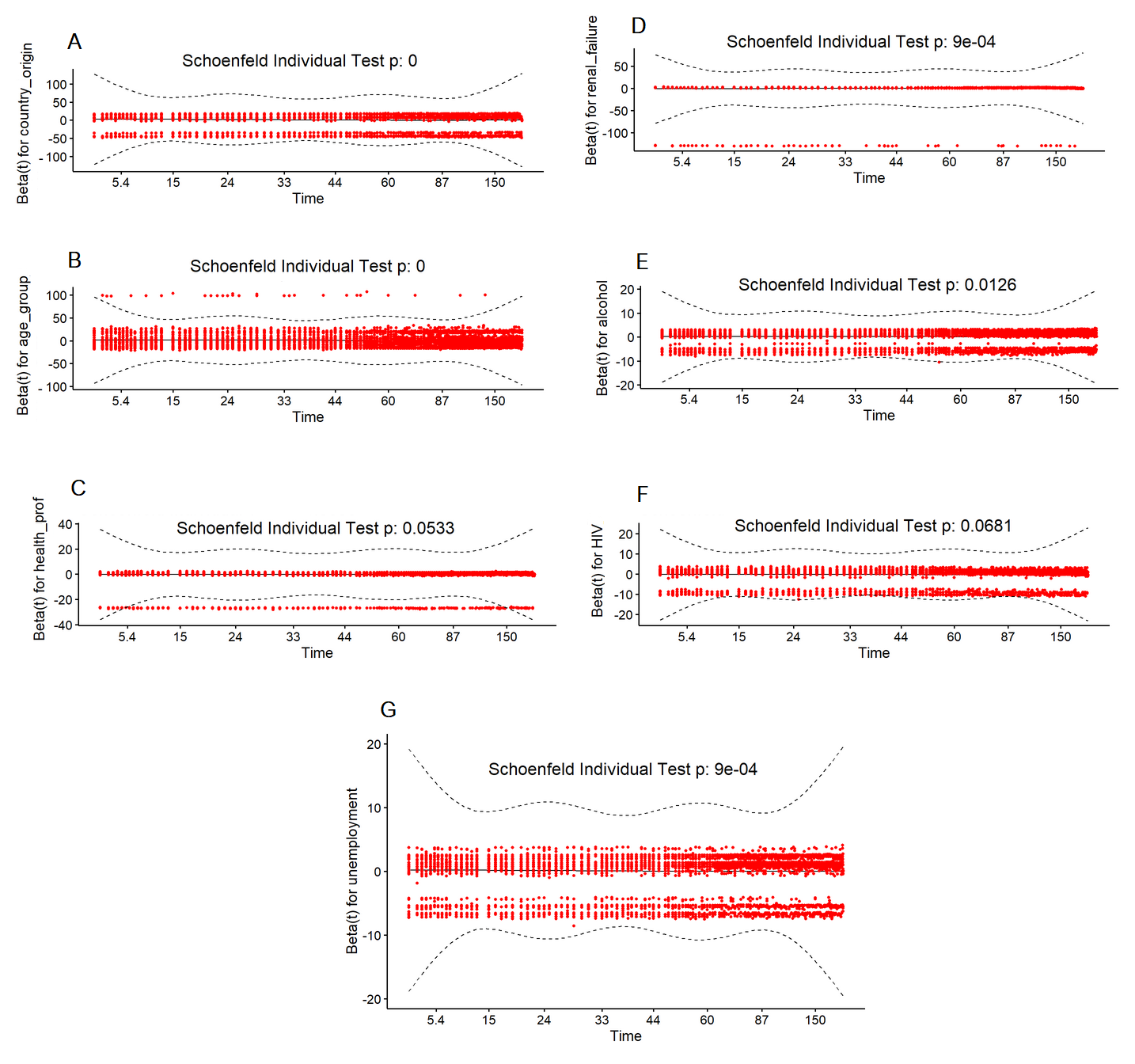

Supplement: Supplementary file 2 — Additional file 2: Supplementary Figure 1. Schoenfeld residues for the variables in the final Cox model - Patient delay. A - Country of origin, B - Age group, C - Health professional, D - Chronic renal failure, E - Alcohol abuse, F - HIV infection, G - Unemployment. [file 12889_2021_12245_MOESM2_ESM.docx]

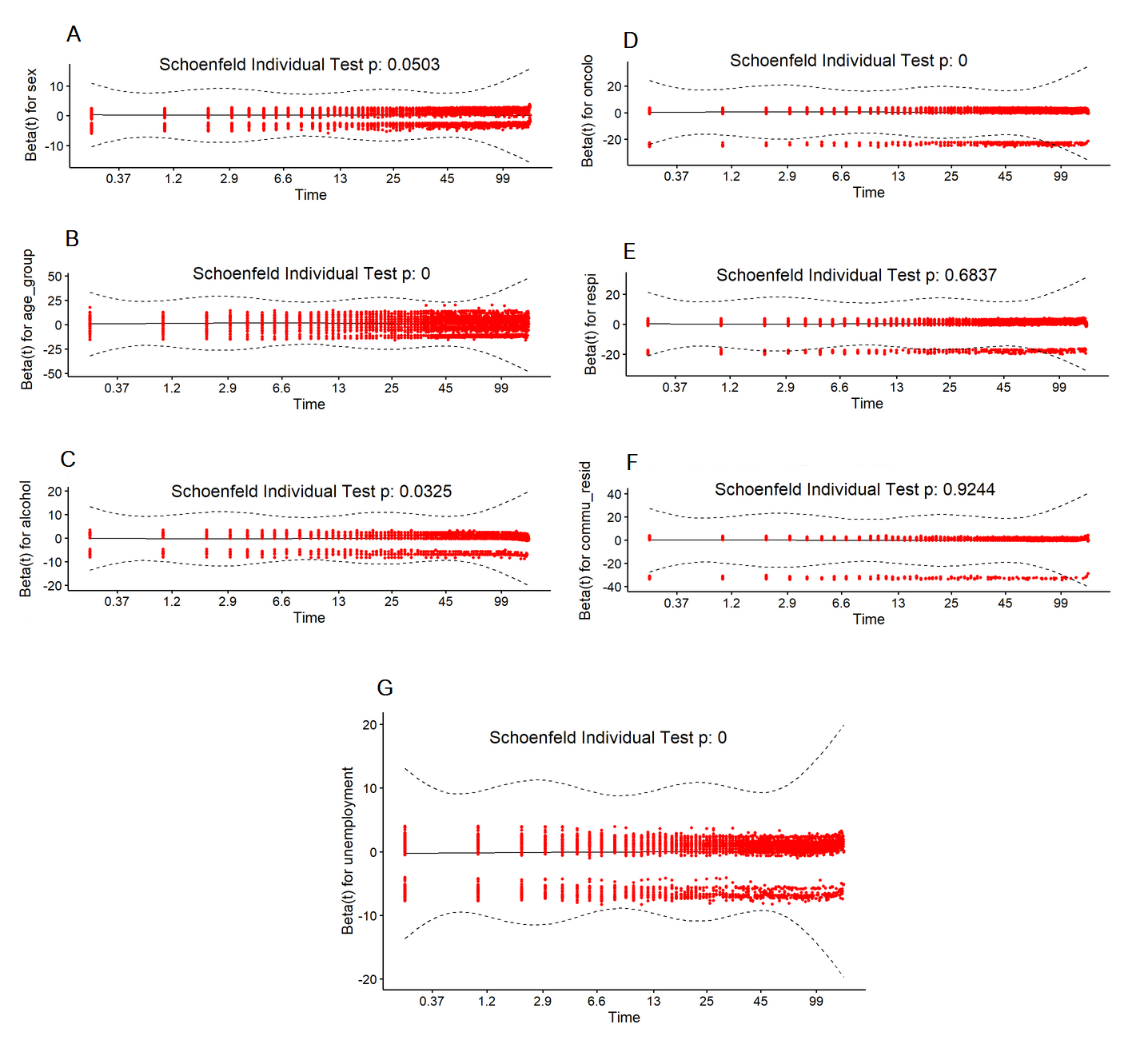

Supplement: Supplementary file 3 — Additional file 3: Supplementary Figure 2. Schoenfeld residues for the variables in the final Cox model - Healthcare delay. A - Sex, B - Age group, C - Alcohol abuse, D - Oncologic diseases, E - Respiratory diseases, F - Community residence, G - Unemployment. [file 12889_2021_12245_MOESM3_ESM.docx]

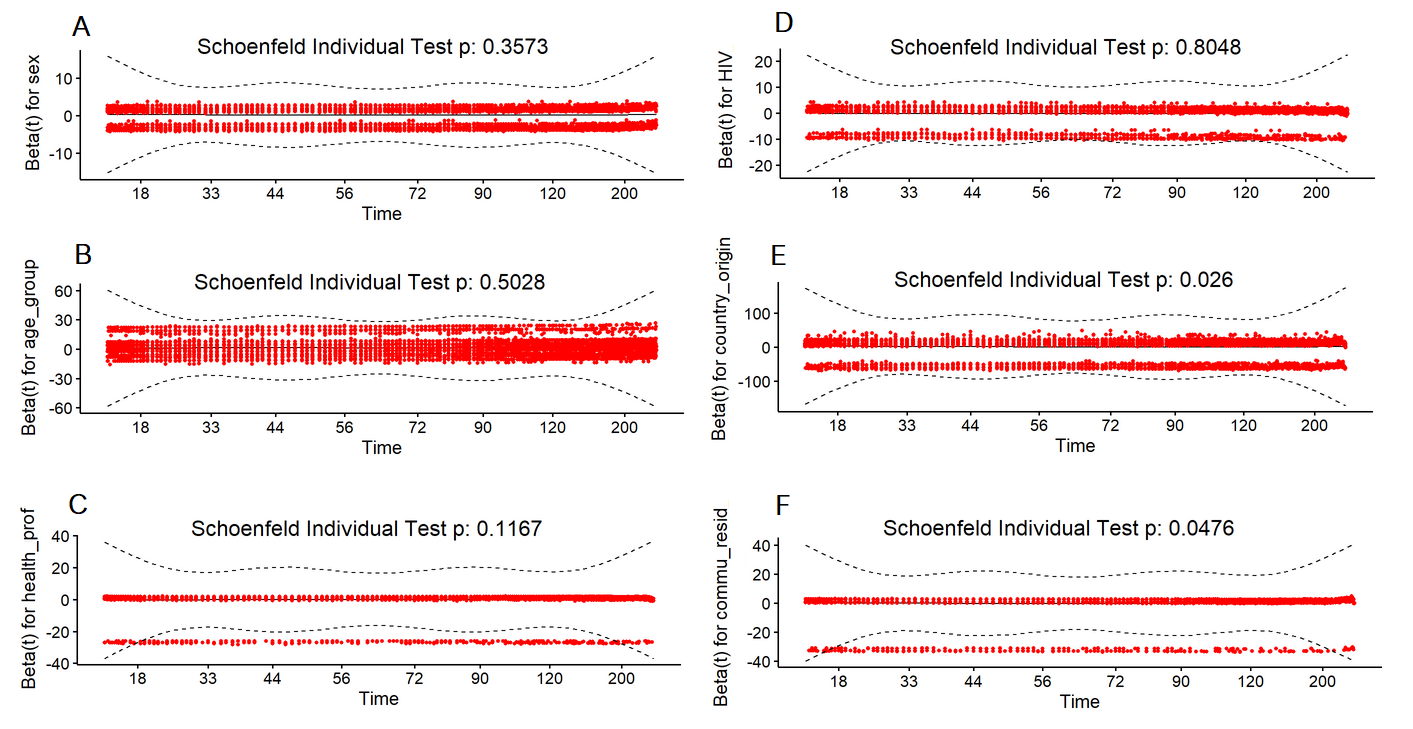

Supplement: Supplementary file 4 — Additional file 4: Supplementary Figure 3. Schoenfeld residues for the variables in the final Cox model - Total delay. A - Sex, B - Age group, C - Health professional, D - HIV infection, E - Country of origin, F - Community residence. [file 12889_2021_12245_MOESM4_ESM.docx]
